# Twelve tips for using synchronous virtual classroom technologies in medical education

**DOI:** 10.15694/mep.2021.000066.1

**Published:** 2021-03-11

**Authors:** Karl Luke

**Affiliations:** 1 Cardiff University

**Keywords:** virtual classrooms, synchronous teaching, online lectures, digital education, online learning, COVID-19

## Abstract

This article was migrated. The article was marked as recommended.

The COVID-19 pandemic has resulted in unprecedented transformations in medical education, with a shift from face-to-face learning activities to digital education. Virtual classroom technologies have played a pivotal role in supporting synchronous teaching activities; however, it can be extremely challenging for many educators to use virtual classrooms tools effectively. This article presents twelve tips based on best practices in educational design, learning theories, current research in online learning, and the authors’ personal experiences of designing online activities within medical education. The twelve tips aim to help medical educators use virtual classroom solutions more effectively to promote learner engagement and learning.

## Introduction

**Figure 1.**
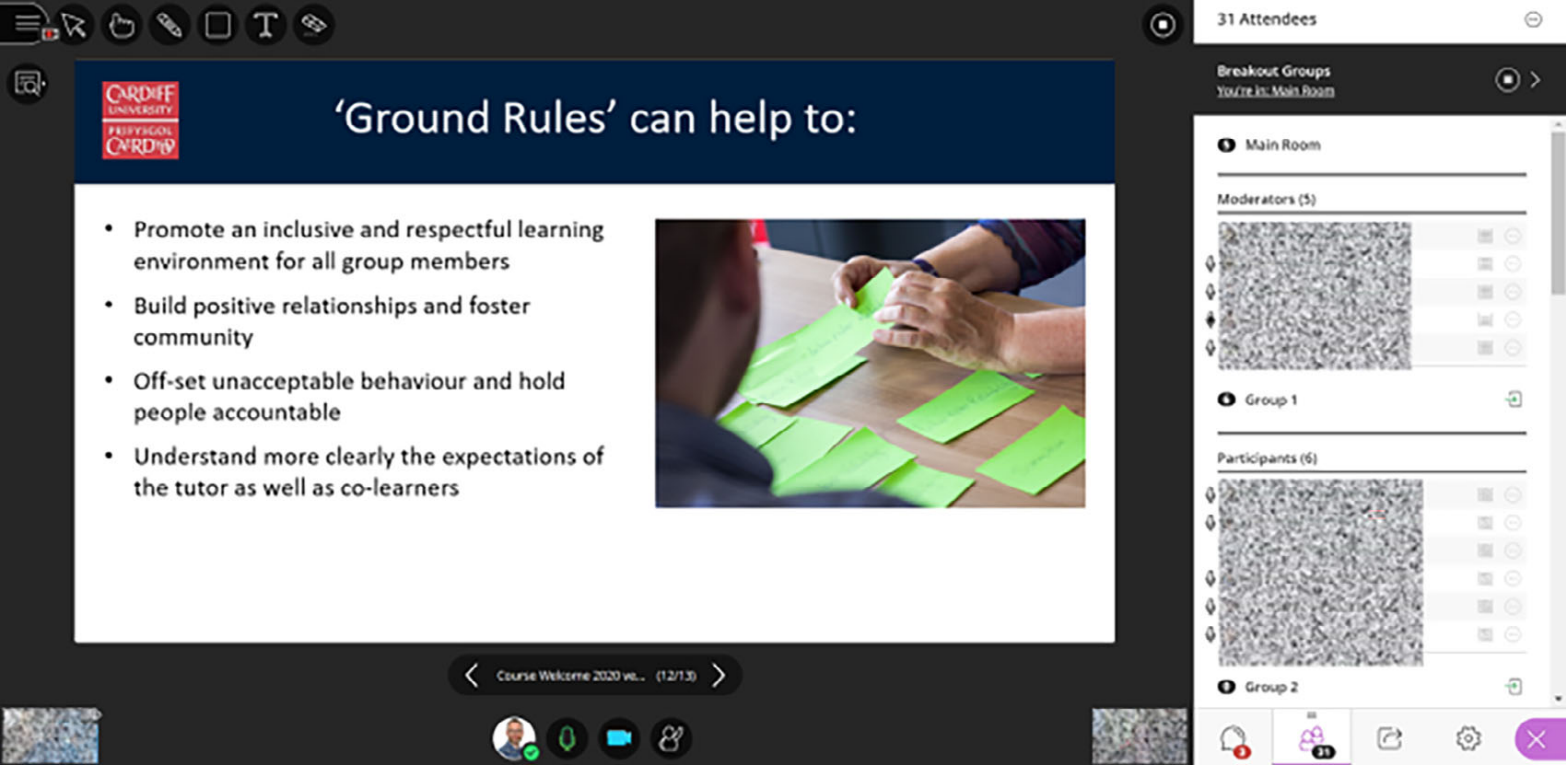
Screen capture of a virtual classroom session, using Blackboard Collaborate (identifiable information pixeled).

The rapid advancements with computer and internet technologies over the past decade have expanded the educational possibilities offered by virtual communication tools to mediate teaching and learning activities. Furthermore, the COVID-19 pandemic has resulted in unprecedented transformations in medical education, with a shift from face-to-face learning activities to digital education. Virtual classroom technologies ( Figure 1) have played a pivotal role in supporting such transformations and have been used to mediate real-time interactions from different places ( Gordon *et al*., 2020; Sandars *et al*., 2020).

Virtual classrooms are defined as a “digital environment...allowing tutors and staff to communicate, interact and engage synchronously in teaching and learning activities” ( QAA, 2020, p. 16). Virtual classrooms are a tool for mediating *synchronous* learning, whereby learners engage with material and instructors in real-time, although not necessarily in the same place. This contrasts to *asynchronous* learning, which does not involve learners in the same place or at the same time (e.g. a task to gather information on a topic individually by a set date).

This article aims to support medical educators in transitioning to online teaching and explores ways to effectively facilitate educational opportunities and increase learner engagement within virtual classrooms. This guide draws upon current research associated with digital education and learning theories and paradigms, including Humanism, Social Constructivism and Cognitivism, to outline twelve practical tips for supporting the effective use of virtual classrooms within medical education. As well as drawing upon previous research undertaken in this area, the article also offers insights from the authors’ decade of experience in designing and facilitating online learning within medical education.

## Tip 1: Align with course objectives and intended learning outcomes

When using virtual classrooms, instructors should carefully set learning objectives, design learning activities, and *constructively align* the use of the technology with the rest of the curriculum ( Biggs, 2011). Educators should consider the purpose of designing synchronous sessions and critically reflect on why a synchronous session is the preferred format over asynchronous delivery. A well-designed online programme should offer a balance between synchronous and asynchronous delivery ( Murphy, Rodríguez‐Manzanares and Barbour, 2011). A synchronous session should offer opportunities for learner interaction, present novel insights, or provide engaging activities, and should avoid duplicating what is covered elsewhere in the curriculum (e.g. in readings, videos, and discussion boards).

Virtual teaching via synchronous technologies may not be suitable in many contexts, such as clinical skills training and professional practice, however, virtual synchronous teaching is seen as appropriate in many medical fields, including anatomy, imaging, physiology, and pathology ( Sidpra *et al*., 2020). Virtual classroom sessions should be relevant in terms of both content and activity and designed to meet learners’ needs. Synchronous sessions should provide additional value to the learners’ learning experience in an online course or programme. For example, sessions could include a brief direct-instruction component, practical demonstration, or provide access to a guest speaker ( Lowenthal, Dunlap and Snelson, 2017).

## Tip 2: Consider the learning design

Virtual classroom technologies can be used to mediate learning experiences in a number of different ways, including:

(1) **Online Lecture with Interactivity.** Closely resembling face-to-face delivery, instructors will be mostly talking and providing didactic instruction. In this scenario, learners are often muted, and the instructor shares a presentation or screen to support their delivery. Sessions can often be recorded for learners to revisit and revise (and for learners who cannot attend). However, in the online environment it is important to build in regular breaks (ideally every 15-20 minutes) for a breakout task or reinforcement activity (e.g. quiz, discussion, Q&A).
(2) **Activity-based Session / Tutorial.** Sessions are based around activity, using breakout rooms to split learners into small groups with discussion-based tasks to work on. Often sessions are bookended with an introduction to the topic and a summary at the end. Plenary feedback can be offered via a ‘main room’ periodically. This can be highly engaging and provide learners with high quality peer interaction. However, it can be more difficult to achieve in larger groups and there is a risk of learners’ attention wandering in breakout rooms. Therefore, it is important to devise highly structured tasks of the appropriate length.
(3) **Question & Answer (Q&A) / Panel Discussion.** These sessions can make effective reinforcement or revision sessions. Learners can submit questions in advance, address them in the session and engage in discussion. This allows issues to be addressed in real-time in a supportive environment and provides the instructor with immediate feedback on learners’ understanding. A nice variation on this learning design is a panel discussion, where colleagues can be invited to provide a short (e.g. 5 minute) presentation on a clinical topic and then participate in a debate or present controversial questions which require learners to ‘pick a side’. When learners are asked to state an opinion, they become more invested in discussing it ( McBrien, Cheng and Jones, 2009). It also gives learners access to a range of voices and can expose them to real academic debate.
(3) **Flipped Classroom.** A workshop style session could mix short presentations, Q&A, and polling / audience response tools, to summarise and reinforce content previously delivered (asynchronously) online. This approach takes pressure and risk off the synchronous session in terms of content delivery, while providing opportunities for active learning. However, this relies on learners engaging with set activities, which might be specifically challenging for some learners. Therefore, it is often useful to include a range of different activities to engage learners ( Moridani, 2007).
(4) **Consultations.** Learners bring challenging dilemmas, clinical case studies or problems and seek the group’s input and advice. This can be particularly effective with adult learners or in project-based courses.

Gordon *et al*. (2020) report that during the COVID-19 pandemic medical educators have used virtual classroom technologies for seminars, debates, team-based learning, simulation sessions, assessment writing workshops, and clinical skills sessions. Gordon *et al*. (2020) also report on clinical placement based learning being facilitated by a blend of online synchronous and asynchronous teaching methods, including videoconferencing and flipped classrooms with Q&A, though there are clear challenges in replicating ‘hands-on’ experiences when delivering clinical skills training remotely. However, Chao, Hung, and Chen (2012) argue that virtual classrooms can mediate synchronous oral assessments and some synchronous practice assessments, whereby educators assess whether learners have acquired the knowledge of component tasks associated with practical skills, such as identifying standard operating procedures (SOPs) in clinical practice or procedural steps associated with a specific clinical skill.

With careful planning virtual classrooms can also be used for both small group and large group teaching. However, there is recent evidence to suggest that smaller group sizes enhance effective collaborative learning in online environments, whereby small group sizes correlate positively with enhancing online group cohesion, efficiency and communicability as well as improving individual learners’ performance ( Saqr, Nouri and Jormanainen, 2019). Educators should therefore critically consider the design of synchronous sessions and design educational opportunities to engage every learner, which will help tackle issues associated with isolation and inactivity.

Importantly, design virtual classroom sessions which are appropriate to the skills, understandings and abilities of the learners and consider their prior knowledge and experience. It is important to recognise that novice learners may benefit from structure and guidance, while experienced learners may benefit from more space and independence ( Kirschner and Hendrick, 2020). While an expert may be able to successfully solve a problem after been taught a certain technique, a novice might benefit from a more structured approach and guidance in using that technique for problem solving. As such, virtual sessions designed on the premise of problem-based learning or discovery learning may actually be detrimental to novice learners. Therefore, if designing curricula using a series of synchronous virtual classroom sessions, design opportunities to scaffold learning appropriately and gradually fade instruction and guidance as the learner progresses ( Kirschner and Hendrick, 2020). Well-designed instruction should ensure learners are not overwhelmed by task complexity and tasks are sequenced from ‘simple’ to ‘complex’ with appropriate support.

## Tip 3: Utilise a tool which is integrated with a Learning Management System

There are numerous online virtual classroom platforms, such as Microsoft Teams, Zoom, Blackboard Collaborate, Adobe Connect and TopHat ( Sidpra *et al*., 2020). If possible, a system that is integrated into a Learning Management System (LMS) should be used. This is recommended as participant enrolments are simplified and it helps organise online learning content in one location, streamlining the educational process for both educators and learners. Using an integrated virtual classroom tool also helps promote the use of the LMS for other collaborative, asynchronous, activities ( Sandars *et al*., 2020). Virtual classroom sessions can be typically recorded and uploaded into the LMS for later viewing by learners who were unable to attend the lecture, had technical issues, or experienced other problems. If it is not possible to use a virtual classroom tool within a LMS, educators should critically consider the security features of the tool they wish to use and ensure it offers features such as a waiting room and password protection. This will ensure that only registered educators and learners are permitted access to the synchronous session and will avoid allowing “trolls” and other disrupters ( Kohnke and Moorhouse, 2020).

## Tip 4: Explore the virtual classroom ‘affordances’

Within many virtual classroom tools there are a range of *affordances* - or action possibilities ( McGrenere and Ho, 2000) - available to the medical educator, which can mediate a range of specific activities and interactions. This is far from an exhaustive list of affordances, but this list highlights some possibilities offered by virtual classroom tools.

**Figure 2.**
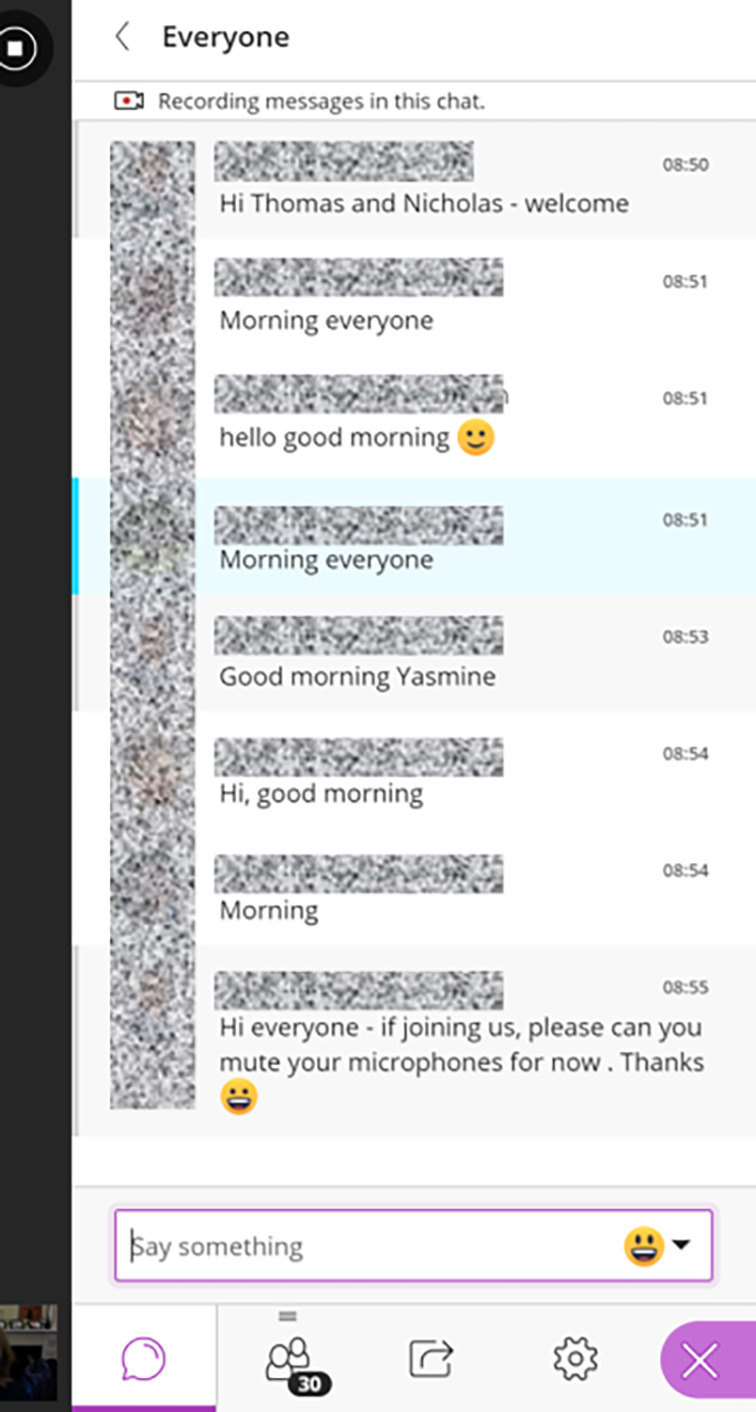
Screen capture of a chat window within Blackboard Collaborate (identifiable information pixeled).

(1) **Text Chat and Emojis.** Text chat tools are available in most platforms and can be used to open a second channel for discussion and debate which can enrich interactions ( Figure 2). Whilst presenting, learners can use the chat to ask questions without interrupting. Learners can use chat to respond or react to content being presented. Learners can also send private text messages to the instructor when they need help. The chat tool is particularly helpful for shy learners who may prefer writing over speaking. Emojis are small digital images or icons used to express an idea or emotion. They can be effective in conveying non-verbal feedback and can help in interacting with learners. For example, learners can use the thumbs up or down emojis to demonstrate agreement or disagreement with a statement or opinion ( Dixson *et al*., 2017).
(2) **Breakout Groups.** Breakout groups allow instructors to split the class into smaller groups and set a task or discussion point, which increases opportunities for learner interaction and participation. In breakout rooms, learners can have discussions, work on projects, role play, or engage in brainstorming sessions. Breakout rooms can help learners socialise and get accustomed and can be beneficial for learners who are uncomfortable participating or speaking up in a large group. Xenos (2018) recommends that best practice is to frequently engage learners in small groups using breakout rooms (at least once in every session) and then gather all learners for discussion and feedback in the main room.
(3) **Polling / Audience Response.** From the basic built-in polling tools available in most virtual classroom platforms to more sophisticated audience response tools (such as Mentimeter), asking questions of learners can be a very powerful way of reinforcing learning, checking understanding or prior knowledge, and stimulating discussion ( Kohnke and Moorhouse, 2020).
(4) **Whiteboard.** The whiteboard can be a collaborate canvas used by instructors and learners to write, draw, or display pre-made content, much like a physical whiteboard or flipchart. Key points can be annotated on the board, or learners can be asked to write their responses to a specific question. Learners can be invited to contribute to the whiteboard as an engagement exercise (e.g. highlight elements on a diagnostic X-ray or collaboratively list risk factors associated with a specific clinical condition).
(4) **Screen Sharing.** Screen sharing is an effective method for displaying a video, slide presentation or pictures. Instructors can also demonstrate how to perform a specific task by sharing their own screen (e.g. how to complete an assessment form or paperwork).

## Tip 5: Use Cameras and microphones appropriately

Educators who wish to actively involve learners within the virtual classroom will need to consider devices, peripherals, and technical configurations ( Peachey, 2017). Where possible, it is recommended that educators and learners wear headsets to reduce extraneous noises, echo, and audio feedback. Educators should ensure that participant microphones are muted when presenting. This will avoid unintended background noise from the learners’ environments.

It is important to respect learners’ privacy and safety within the online environment. Whilst educators may encourage learners to use cameras and/or microphones, these should not be mandated. Some instructors might insist that all learners enable their cameras, however, it is recommended that learners are offered a choice whether to turn on their video or use virtual background filters. Learners might be uncomfortable with displaying their living space with peers or may not want their images captured for privacy and safety reasons. Also, some learners might have unreliable internet access and low bandwidth. The context and objectives of the virtual classroom are also worth considering in relation to the use of cameras. For example, a presentation from a guest speaker or a ‘journal club’ session, whereby learners verbally discuss a topical article, may not require participant cameras. However, a peer consultation activity, or a practical session on communication skills, may be enhanced by encouraging participants to enable their cameras. To enhance the social presence of a session, educators can encourage learners to upload profile pictures which can be displayed when a video source is disabled. To help assess attention and engagement, real-time check-in activities or interactions can be used, for example text chat, polls, or emojis.

## Tip 6: Design for interaction and engagement

It is important to design opportunities for peer interaction within the virtual classroom ( Peachey, 2017). It is important to stress that virtual classrooms should not be used solely for didactic presentation, whereby an asynchronous video would be more appropriate. To increase interaction in virtual classrooms, it is recommended that educators should: provide both informal and structured time for peer interaction; actively involve learners during the sessions, for example collaborating on a response to a clinical problem or peer review each other’s work; and invite questions from learners unable to attend and respond to these questions during the live session ( Lowenthal, Dunlap and Snelson, 2017).

It is recommended that educators break the session into a series of components, interspersed with small learning activities ( Kirschner and Hendrick, 2020). For example, ask learners questions and/or use the chat function and polls to add interactivity. Throughout the session, encourage learners to use the text chat function and non-verbal cue functionality (e.g. emojis, thumbs-up, hand-raise) to promote interactivity and obtain learner feedback. The challenge here is ensuring that the educator and learners are not distracted by the text chat but can also monitor interactions. If possible, teaching in pairs can be effective, with one colleague presenting and one colleague moderating the text discussion.

By their very nature, virtual classroom sessions can also be rather teacher-centred compared with face-to-face sessions and initial cohort discussions may be characterised by periods of silences and shorter learner responses ( Moorhouse, 2020). When asking questions, it is important to give learners sufficient time to prepare/write their answers in the chat. Posing questions throughout the session can be an effective strategy for maintaining engagement but provide ample time for learners to process the questions and formulate responses. An alternative approach is to pose a thought-provoking, relevant question and give learners a few minutes to write down their thoughts/answers. This allows learners to prepare responses without being put on the spot.

To increase opportunities for interaction and engagement, consider starting each session with an ice-breaker activity and/or ‘get-to-know-you’ task ( Christopher, 2014). This will help foster collaboration and relationship building and provide opportunities for learners to familiarise themselves with the technology before undertaking course-oriented activities ( Dixon, Crooks and Henry, 2006).

## Tip 7: Consider length and take breaks

Currently, there is little evidence available which evaluates the optimal duration for a virtual classroom session. However, Cognitive Load Theory (CLT) and ideas associated with information processing emphasises that when information is presented that exceeds learners’ cognitive capacity, this results in cognitive overload, which has a detrimental impact on learning ( Sweller, 1988; Mayer, 2005). There is also a concern associated with the phenomenon of ‘screen fatigue’ for both educators and learners ( Kohnke and Moorhouse, 2020; Lemov, 2020). Evidence-based guidelines associated with the design of asynchronous video content stresses that the duration of video content should be limited to maintain learners’ attention and engagement and longer videos should segmented into ‘chucks’ with interactive elements ( Dong and Goh, 2015; Brame, 2016).

Following these recommendations, shorter virtual classroom sessions may be optimal, and sessions should be structured to facilitate learners’ ability to integrate information with relevant prior knowledge ( Dong and Goh, 2015). Clearly segment the session and clearly communicate the session structure to learners at the start of the event, which will help learners to organise content into coherent cognitive structures ( Mayer, 2005). Providing worked examples, which demonstrate how to solve a specific problem or modelling expected outcomes, can be effective teaching strategies for optimising germane load ( Collins *et al*., 2020). During live sessions, instructors may like to experiment with embodied techniques and encourage learners to stand up, stretch, walk around, and engage with others ( Vuuren and Freisleben, 2020). Encouraging such techniques might prove to be particularly effective in synchronous sessions centred around role-playing a clinical scenario, undertaking a peer-led consultation activity, or engaging in a panel debate.

In the online environment it is important to reduce extraneous information such as decorative images and multiple application windows ( Collins *et al*., 2020). Try to build regular breaks (ideally every 15-20 minutes) for a breakout task or reinforcement activity (e.g. quiz, discussion, Q&A). If the functionality exists, advise learners to use an ‘Away’ status during breaks to indicate their temporary absence from the session. Prompt learners to remove the “Away” status as soon as they are back, which helps with (re)starting the session.

## Tip 8: Develop guidelines for learners and manage expectations

Importantly, engaging with instructors and peers in a virtual classroom may be a new way of learning for many. Clear guidance should be developed to enhance learners’ metacognition skills and self-efficacy, as well as support learners’ ability to effectively engage and contribute within a virtual environment ( Kirschner and Hendrick, 2020). Carefully orient learners to the notion of collaborative learning and facilitate opportunities for collaboration throughout ( Jennings, 2020). It is also worthwhile adjusting expectations of having the same duration and frequency of sessions as face-to-face class schedules. Keeping things shorter will help reduce cognitive load and give learners the space they need for processing and recharging ( Mayer, 2005).

Avoid assumptions relating to the digital literacy skills of learners and provide sufficient guidance in advance, explaining how to use the virtual classroom tool and how to configure devices appropriately. Providing learners with a short recording of a virtual classroom session can be effective in demonstrating how sessions operate and highlights what learners can expect in advance of participating in a live session ( Lowenthal, Dunlap and Snelson, 2017). Educators can also work in active partnership with learners to co-design explanatory guidance and materials ( Evans and Luke, 2020). If undertaking a series of virtual classroom sessions, offering a practice session in advance may provide a good opportunity to check-in on learners, explore any technical issues and begin rapport building. A practice session may also help reduce any learner anxieties, particularly for those who are new to engaging in virtual classrooms. Learners can also co-design ‘ground rules’ - or a ‘charter’ - for interacting with each other during live sessions ( Jennings, 2020). Ground rules typically cover aspects such as communication etiquette, team leadership, expectations of contributions, and how to resolve conflicts, and are useful in agreeing both educator and peer responsibilities and managing expectations.

Informing learners of what to expect is also important and communications should be effective and consistent. Select a specific communication method and continue with this method, for example email, LMS announcement, or embedded message within asynchronous video lectures. Contact learnersbefore each synchronous session and inform them of the session content, how they should prepare, and what they will be expected to do within the live session. Be as concrete and specific as possible; when learners have time to prepare, they are often more invested in the discussion and willing to participate ( Kaufmann and Vallade, 2020). Ensure expectations of the session are clearly articulated, including how learners interact, and set clear expectations for interaction with the instructor and their peers (e.g. chat, Q & A, via microphones, use of break out rooms). Information from learners can also be collected in advance which will assist in preparing appropriate questions and materials. This activity could be conducted via a short quiz before the session to assess prior knowledge of a topic. It will also demonstrate to learners that the educator is actively interested in their progress, which will help support interaction in the synchronous environment.

## Tip 9: Humanise the experience and offer flexibility

The adoption of humanistic values within medical education - such as demonstrating honesty, empathy, and compassion, building personal connections, and nurturing dignity, respect, and confidentiality - can benefit medical learners, clinicians, and patients ( Cohen and Sherif, 2014). Humanism also has important implications for online learning, as it encourages educators to consider affective domains and learner motivations when designing content and facilitating learning ( Arghode, Brieger and McLean, 2017). Developing an engaging social presence in the online classroom and humanising the sessions are key parts of developing an effective online community ( Collins *et al*., 2020; Jennings, 2020). Moreover, incorporating humanistic principles within virtual classrooms can provide learners with the conceptual foundation and skills for humanistic clinical practice ( Cohen and Sherif, 2014).

Research demonstrates that the characteristics, personality, presence, and behaviour of the instructor directly shapes learners’ online interaction and engagement ( Shoepe *et al*., 2020). To promote learner engagement, adjust the tone of written materials and communications to be more conversational when appropriate. Academically rigorous language can come across as rigid or impersonal online. Performing a social check-in at the beginning of the virtual class can also be an effective strategy to promote engagement. Whilst learners are arriving into the session, use this as an opportunity to socialise and chat, for example discuss topical events or ask questions about the learners’ lifeworlds. Consider displaying a welcome slide featuring a news item, cartoon, or trivia question to spark conversation before class formally starts. This helps break down social barriers while creating the expectation of interaction. At the start of the session, clearly situate the session within the wider module and conclude by reminding learners of the programme schedule. Communicating with learners in an online environment may take additional planning and effort compared with face-to-face instruction. However, scheduling time for building rapport and communicating effectively is correlated with successful learner outcomes in online education ( Glazier, 2016).

Be flexible and forgiving regarding attendance requirements and offer alternatives whenever possible for learners who are unable to attend or may need to leave early/arrive late. While it is good practice to record sessions, recordings are not the same as being there in real-time. Therefore, consider some other ways to have learners engage with content and each other asynchronously, such as informal groups via mobile messaging apps or discussion forums. Consider developing specific activities or meaningful questions/prompts for asynchronous learners to explore whist watching the recording to promote generative learning ( Fiorella and Mayer, 2016).

## Tip 10: Design opportunities for feedback and encouragement

Research into digital education highlights the importance of timely and formative feedback in fostering learning in online environments ( Castro and Tumibay, 2019). It is essential that online learners constantly receive timely feedback on their performance and understandings from the educator, which also helps keep learners engaged and motivated ( Jennings, 2020; Reyna, 2020). Within the virtual classroom formative feedback can be offered based on individual activity (e.g. after undertaking a class poll to test understandings) or collaborative tasks (e.g. collaboratively designing a patient management plan). Feedback can also be instructor or peer-led. For example, after a breakout room activity schedule time for both instructor and peer feedback and debriefs. When facilitating formative feedback, based on group or individual activity, ensure feedback is timely, honest, direct, and constructive ( Tanis, 2020). Recommend concrete steps for improvement and signpost additional resources and services which can be accessed flexibly.

Social presence theory suggests that the level of social presence and immediacy of synchronous interactions influences learner engagement and performance ( Bickle, Hirudayaraj and Doyle, 2019; Dahlstrom-Hakki, Alstad and Banerjee, 2020). Supportive communication and developing constructive relationships with learners are important aspects in the online environment. When providing feedback, educators should be mindful of negative feedback, as online learners are easily discouraged and prone to demotivation ( Peachey, 2017). It is also important to recognise that learners will assume various roles within virtual classrooms, with some offering more participation than others ( Macnaught and Yates, 2020). Within an online community some learners may engage with *legitimate* *peripheral* *participation* ( Lave and Wenger, 1991), whereby learners initially undertake passive activities, such as observing senior members and reacting to them, before undertaking more complex tasks that require more responsibility. Educators should design opportunities which actively encourage all learners to meaningfully participate in the virtual classroom. At appropriate times, educators should also be willing to give up control of the direction or content of the session and assume a participant role, thereby encouraging learners to take control of the discussion ( Palloff and Pratt, 2003).

## Tip 11: Integrate evaluation

Medical educators collect evaluation data for a variety of reasons: to gather ideas for ongoing revision of a programme; to appraise the effectiveness of new educational initiatives, tools, resources, or innovations; to document that programme objectives have been met; and to flag up ineffective training and enable educators to make changes for improvement ( Goldie, 2006). The evaluation of virtual classroom teaching is an important activity which should be considered within the design and implementation of virtual classrooms ( Christopher, 2014).

Educators should consider how they will systematically collect data to inform future improvements. This can come in the form of post-session qualitative methods, such as interviews, focus groups, or survey collection ( O’Flaherty and Laws, 2014). Virtual classroom tools may also be integrated with a LMS, which may also provide educator access to quantitative performance data via learning analytics and educational dashboards ( Boscardin *et al*., 2018). However, many virtual classroom tools offer features which can provide insightful qualitative evaluation from learners *during* the teaching session. The polling features, available within many virtual classroom tools, offer opportunities for educators to gather real-time data and track learners’ feedback. Polls can be quickly deployed for timely evaluation and anonymous data can be gathered regarding the content or structure of the sessions. Such data can provide important insights regarding how to enhance sessions and allows opportunities to implement iterative improvements to support learners ( O’Flaherty and Laws, 2014). Rapid and iterative evaluation is especially useful when designing a series of virtual classroom sessions, whereby data can inform decisions and strategies throughout the programme (e.g. topic material to revisit, specific case studies to discuss, or facilitation techniques to modify).

## Tip 12: Consider barriers

Learners can face a number of challenges with online learning, including a sense of isolation, the need for self-discipline, and developing digital literacy, whereby learners who lack digital literacy skills might be disadvantaged ( Peachey, 2017). To address these issues, try to keep membership of virtual classrooms consistent so learners have an opportunity to form relationships and emotional attachments over a period of time. Supplement live sessions with resources learners can explore independently and consider posting summaries of major discussion points through LMS announcements or email. To help support inclusivity, avoid or explain culturally specific references and use inclusive language throughout the session to ensure that stereotyping is not present ( Gishen and Lokugamage, 2019; Sanger, 2020).

Consider potential barriers to engagement such as different time zones and technical issues. Synchronous activity could disadvantage some learners due to lack of access to hardware, insufficient software, or inconsistent access to the internet. Review the flexibility of the curriculum timetable and critically consider if the timing of the teaching and learning activities might negatively impact on some learners (e.g. consider religious or cultural holidays and celebrations). Using an online diversity and inclusion calendar can be helpful in planning activities. Educators should strategically schedule live sessions - they do not need to be scheduled weekly - and vary the day/time and provide alternating schedule patterns ( Lowenthal, Dunlap and Snelson, 2017). Online scheduling tools, such as Doodle, can be useful in collaboratively determining a schedule. As discussed, alternative opportunities for learners to engage outside of the virtual classroom should also be provided (e.g. pose questions before/after session in discussion boards and record the virtual sessions).

While many learners with disabilities highly value synchronous discussions ( Dahlstrom-Hakki, Alstad and Banerjee, 2020), fully live sessions might not be as accessible to all learners with disabilities. To help design inclusive virtual classrooms, resources should be provided in advance, which could resemble an outline and any supporting materials (e.g. PowerPoint files, articles, handouts). Such resources can be downloaded and converted to alternative formats (e.g. audio file or braille) and can help learners identify areas of focus during the session. During the session use a steady pace to deliver content; learners using screen readers will need to balance listening to the screen reader to hear the content and listening to the speaker. It is also important to ensure the technology is compatible with assistive technology. Learners using specialised keyboard may be limited in their participation, therefore provide as much information in advance as possible so learners can prepare comments and questions. Be mindful that navigation between different tools without a mouse and typing can take time and allow sufficient time for learners to process information and respond to questions. Offer learners the option of contributing verbally if they prefer this method to writing in the chat or Q&A. If chat and Q&A is used, emphasise that learners’ contribution is more important than spelling or typing accuracy.

## Conclusion

Medical education involves a complex interplay and dependence between didactic learning and clinically relevant material ( Johnson *et al*., 2013). Medical educators also need to develop and implement innovative solutions in response to the challenges facing global society and future trends in the use of interactive technologies ( Goh and Sandars, 2020). Within medical education, virtual classroom technologies can be effective tools for enabling educators to deliver teaching at a distance whilst maintaining social connectivity. If used appropriately, virtual classrooms technologies can meditate interactive content, educator presence, and timely and formative feedback, which are all seen as important ingredients in online learning ( Castro and Tumibay, 2019).

While virtual classrooms can be an important part of most module designs, they should not be over-relied upon for teaching. Remember that online sessions compete with the real-world distractions of the environment that learners are studying in, so maintaining engagement is vital ( Luke, 2020). Synchronous activities might also disadvantage some learners, such as those with specific disabilities, limited technological literacy, or inconsistent online access, which will require additional consideration and support. Therefore, synchronous sessions should be supplemented with asynchronous content and interactions. However, with careful design and attention to the twelve tips outlined in this article, virtual classroom technologies can provide effective opportunities to mediate real-time communication and collaboration between educators and diverse learners that might otherwise not be possible.

## Take Home Messages

• Carefully consider the learning design of online synchronous sessions and the affordances available within the virtual classroom tool.
• Employ effective teaching and facilitation strategies, grounded in learning theory, which support peer interaction and collaborative engagement.
• Respect learners’ privacy and safety needs. Maintain compassion and remember learners may be transitioning to online learning for the first time.
• Consider potential barriers to engagement, such as different time zones, digital literacy, and technical issues. Live sessions might not be as accessible to some learners with disabilities.

## Notes On Contributors

**Karl Luke** is a lecturer in Medical Education at Cardiff University. Karl is a Senior Fellow of the Higher Education Academy (SFHEA) and Certified Member of the Association of Learning Technology (CMALT). Karl has over 15 years’ experience of designing for teaching and learning in a digital age. Karl’s research interests include digital education, multimodality and sociomateriality. ORCiD ID: https://orcid.org/0000-0002-7765-6126
