# Supplementary material for: Twelve tips for using synchronous virtual classroom technologies in medical education
Source: MedEdPublish (2016). 2021 Mar 11;10:66. [Version 1] doi: 10.15694/mep.2021.000066.1 (PMC10939628; doi:10.15694/mep.2021.000066.1)
Supplement: Peer review history

# Reviewer report: Reviewer response for version 1

**DOI:** 10.21956/mep.18894.r26986

**Published:** 2021-07-09

**Authors:** John Sandars

**Affiliations:** 1 Edge Hill University

This review has been migrated. The reviewer awarded 4 stars out of 5 These 12 Tips will provide an excellent practical guide for all medical educators that is based on the author's practical experience and supported by some key references. It is a large topic but overall, a great summary.

Reviewer Expertise:

NA

No decision status is available

# Reviewer report: Reviewer response for version 1

**DOI:** 10.21956/mep.18894.r26985

**Published:** 2021-06-09

**Authors:** Ken Masters

**Affiliations:** 1 Sultan Qaboos University

This review has been migrated. The reviewer awarded 3 stars out of 5 A useful 12 tips paper on using synchronous virtual classroom technologies in medical education.Although quite a few papers have already been written on the topic (in response to the move to online education because of Covid-19), this paper is a nice blend of theory, practice, others’ and the author's own experience.Although the paper does have great value, I would like to have seen the ideas balanced by a careful awareness of problems and realistic expectations. For example, breakout rooms are a great idea, but readers need to be aware that making them successful takes a lot of hard work, and often, the students will take it as an opportunity to do nothing. A single instructor cannot adequately monitor 50 breakout rooms. The danger with not alerting the reader to this is that the novice (the person who would benefit most from such a paper), is left wondering at their inadequacy, because “the literature” says that these things can be done, but, when they try it, it fails miserably. Worse, well-meaning line managers place pressure on the instructor to “get creative and have interactivity”, and, then these are tried and fail, then the instructor has to give an explanation of why they were unable to reproduce the results that others are getting.My comment above could apply to almost every tip in the paper. So, I really would like the author to go through each tip and add even a few lines, a note of caution about the difficulties and/or limitations (solutions may not be possible) and temper expectations of what is realistic.I look forward to Version 2 of this paper in which this is addressed.

Reviewer Expertise:

NA

No decision status is available

# Reviewer report: Reviewer response for version 1

**DOI:** 10.21956/mep.18894.r26984

**Published:** 2021-03-12

**Authors:** Elisabeth Schlegel

**Affiliations:** 1 Donald and Barbara Zucker School of Medicine at Hofstra/Northwell

This review has been migrated. The reviewer awarded 4 stars out of 5 Thank you for this practical, best-practice resource. I also appreciate highlighting creating an inclusive environment.

Reviewer Expertise:

NA

No decision status is available

# Reviewer report: Reviewer response for version 1

**DOI:** 10.21956/mep.18894.r26983

**Published:** 2021-03-11

**Authors:** Virginia Randall

**Affiliations:** 1 Uniformed Services University of the Health Sciences

This review has been migrated. The reviewer awarded 4 stars out of 5 I enjoyed this clearly written summary of tips for synchronous virtual classrooms. I suspect many medical educators have found or developed similar tips for themselves. I did appreciate the emphasis on humanistic treatment of learners, and the discussion of learners with disabilities.

Reviewer Expertise:

NA

No decision status is available
